# Robust robot image classification toward cyber-physical system-based closed-loop package design evaluation

**DOI:** 10.3389/fnbot.2022.1083835

**Published:** 2023-01-11

**Authors:** Shacheng Liu

**Affiliations:** Hunan Institute of Science and Technology, Yueyang, China

**Keywords:** robust classification, robot image classification, noisy labels, total variation regularization, package design, cyber-physical systems

## Abstract

The package design can transmit the value of a product to consumers visually and can therefore influence the consumers' decisions. The traditional package design is an open-loop process in which a design can only be evaluated after the products are sent to the market. Thus, the designers cannot refine the design without any helpful advice. In this paper, a robust robot image classification is proposed to help the designers to evaluate their package design and improve their design in a closed-loop process, which is essentially the establishment of a cyber-physical system for the package design. The robust robot image classification adopts the total variation regularization, which ensures that the proposed robot image classification can give the right answers even if it is trained by noisy labels. The robustness against noisy labels is emphasized here since the historical data set of package design evaluations may have some false labels that can be equivalently regarded as disturbed labels from the true labels by noises. To validate the effectiveness of the proposed robot image classification method, experimental data-based validations have been implemented. The results show that the proposed method exhibits much better accuracy in classification compared to the traditional training method when noisy labels are used for the training process.

## 1. Introduction

### 1.1. Background and motivations

**Figure 1:**
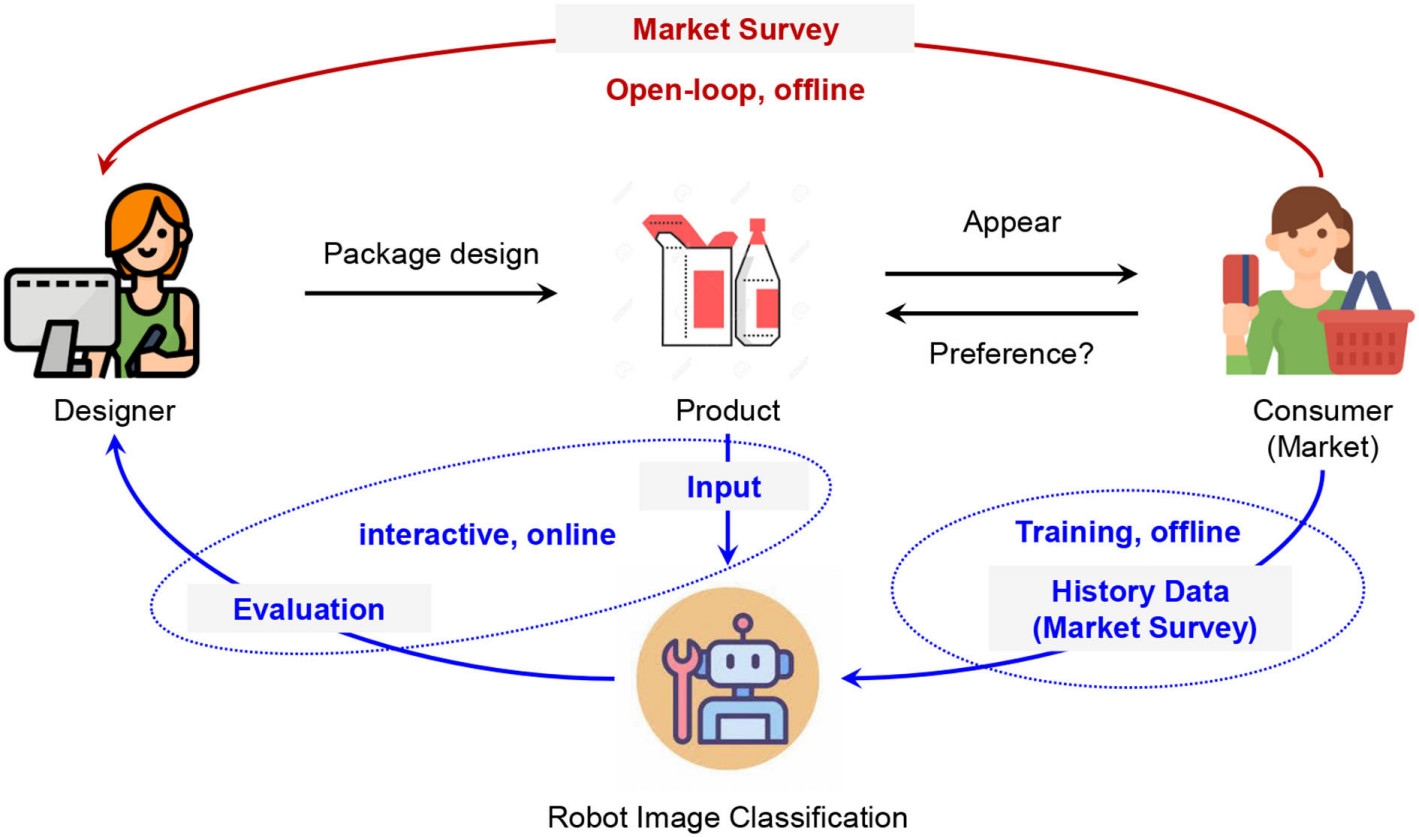
Comparison of the processes of traditional open-loop package design and robot-assisted interactive package design.

A product's value is first transmitted to the consumers by package design visually. A good design can significantly improve the sale performance of a product. Therefore, it is important for a designer to provide a design from the consumers' view of consumption. As shown in Figure 1, the traditional package design is an open-loop process. A designer must figure out the consumers' preferences according to the market survey. The evaluation of the package design for the products can only be implemented after sending the product to the market and obtaining the results of the market survey. The designer cannot refine the design with any helpful evaluation which reflects the preferences during the design process. This hurts the efficiency of the design and also brings risk to the sale performance. Robot image classification makes the interactive package design possible. In robot-assisted interactive package design, a robot image classifier can give an evaluation of the design. The evaluation can reflect the market preferences since the classification models are trained by the historical data of the market survey. Deep learning models have been applied to improve the package design evaluation performance (Shi, 2022). According to Zhao et al. (2018), a graphic design-based model is developed to predict the score of a design. According to Jolly et al. (2018), the conventional neural network has been used to classify a type of design. Definitely, the performance will be good with deep learning models if the labels are correctly given. However, there are lots of unclear or incorrect labels in the data set of market surveys (Xia et al., 2022), which can be regarded as noisy labels. Noisy labels can cause severe over-fitting issues in deep learning models. It is very important to improve the robustness of deep learning-based robot image classification for package design evaluation toward noisy labels.

### 1.2. Related research

Robot classification for package design aims to enable robots or computers to make esthetic decisions about images of the package design in a way of imitating human vision and esthetic thinking (Zhang et al., 2022). The image esthetic quality assessment methods can be categorized into two main streams. The traditional esthetic quality assessment is based on artificial design features. Nowadays, deep learning-based esthetic assessment methods are becoming popular.

In the traditional method of evaluating esthetic quality based on features of artificial designs, the image esthetics are assessed by the two-layer design by an expert. The lower layer has visual features and the higher layer has composition esthetic features. Images can be categorized into high and low esthetics by methods such as support vector machine, which has color matching, the contrast of images, and other features as inputs (Kumar et al., 2019; Wu et al., 2021).

In recent years, with the development of deep learning models, many researchers have started to apply convolutional neural networks to the tasks of image esthetic assessment. For example, according to Wang et al. (2019), convolutional neural networks have been used to specify some high-level abstract features from big data of the image. Furthermore, the structure of the convolutional neural networks is adapted for image esthetic assessment by Darmawahyuni et al. (2019). However, the above methods assume that the used data for training are clean. However, according to the results of Xia et al. (2022), the classification based on esthetic assessment is different from the classification from the viewpoint of the consumers. The labels that reflect the viewpoint of the consumers can be obtained from the market survey. However, the labels obtained from the market survey may suffer the issue of having uncertainties, which makes the labels noisy. It is important to consider how to improve the accuracy in the establishment of robot image classification for package design when using noisy labels for training.

The classification with noisy labels was first addressed by Angluin and Laird (1988) and has just become a very hot topic in the machine learning community (Goodfellow et al., 2016). The first model for noisy labels, proposed by Angluin and Laird (1988) and Rooyen et al. (2015), is for binary classification, which is named the noise model of random classification. The random classification noise model has been extended to the noise model conditioned on classes in Natarajan et al. (2013), which is also known as the class-conditional noise model. The multi-class case has been developed in recent years (Goldberge and Ben-Reuven, 2017; Patrini et al., 2017). The research on classification with noisy labels has the following main streams:

- For class-conditional noise models, one popular method is to adopt robust loss functions to alleviate the issue caused by noisy labels (Ghosh et al., 2017; Feng et al., 2020; Lyu and Tsang, 2020; Ma et al., 2020). The method using robust loss functions works well under simple noises. When the noise is complex and with the high rate, the method performs very poorly.
- Another kind of method tries to improve the robust against label noise by sampling methods (Malach and Shalev-Shwartz, 2017; Han et al., 2018; Wei et al., 2020). In the sampling process, some samples are rejected to improve the robustness, which can be regarded as a semi-supervised learning process (Nguyen et al., 2020). The sampling methods suffer high computational cost and high model complexity.
- The third kind of method is based on estimating the noise transition matrix based on the assumption of the existence of anchor points (Liu and Tao, 2015; Patrini et al., 2017; Yu et al., 2018). The transition matrix is identifiable only if anchor points exist in all classes. When the transition matrix is obtained, the probability of the true labels can be recovered.

### 1.3. Contributions of this article

This paper extends the noise transition matrix estimation method into the robot image classification and proposes a robust robot image classification method for package design evaluation. The main contributions of this article are as follows:

- We adopt a problem formulation of the total variation regularization. The solution to the problem of total variation regularization is consistent with the real transition matrix and the probability distribution of the true labels.
- The stochastic gradient descent can be used to approach the solution that recovers the probability distribution of the true labels from the noisy labels.
- For the first time, a noisy label-aware package design evaluator can be established to help the designer improve their compositions in a closed-loop way.

The research of this article is a big step to establish a computer-vision cyber-physic system for package design evaluation since the issues raised by the noisy labels in the training process of a robot image classifier are resolved.

## 2. Problem description

Let x∈X be a package design composition. Let y∈Y={1,...,C} be the discrete score of the package design, which reflects the consumers' view toward the design. Note that X and Y represent the design space and score space, respectively. A high score of *y* means a high satisfaction with the design. The positive integer *C* represents the highest score. Note that the evaluation is stochastic which obeys a true probability distribution


(1) $$\begin{array}{l} p_{\text{true}}\left(y|x\right):=\text{Pr}\left\{y|x\right\}, \end{array}$$

for every x∈X, different consumers have different opinions. Let ℙ(Y) be the set of all possible probability measures defined on Y. Let $E_{\text{true}}:X\to \mathbb{P}\left(Y\right)$ be the underlying function that outputs the probability distribution of true score by the consumer for a given design x∈X, namely, $E_{\text{true}}=p_{\text{true}}\left(y|x\right)$. In this article, we call $E_{\text{true}}\left(\cdot \right)$ a real evaluator. Essentially, the evaluator $E_{\text{true}}\left(\cdot \right)$ can be regarded as a classifier that gives a label of class y∈Y to each design. Although the basic function in mathematics is the same, we adopt the terminology “evaluator” here since there is an order that larger *y* means a better design.

Note that the real evaluator is unknown. It is necessary to construct an estimated evaluator from the available data set $D_{T}={\left\{x\left(t\right),\tilde{y}\left(t\right)\right\}}_{t=1,...,T}$. Here, we use $\tilde{y}$(*t*) to denote the noisy score of the design *x*(*t*). The noisy score $\tilde{y}$(*t*) represents the randomness in the process of collecting data by market survey. For the extraction of the design *x*(*t*), the following assumption holds throughout the rest of the paper.

**Assumption 1**. *The design x*(*t*) *is extracted identically and independently from the design space*.

Although there is a true *y*(*t*) for every *x*(*t*), the observed score $\tilde{y}$(*t*) is disturbed by noise. Here, we only consider the class-conditional noise. By adopting the class-conditional noise model, the following assumption on $\tilde{y}$(*t*) holds throughout the paper.

**Assumption 2**. *The noisy score $\tilde{y}$*(*t*) *does not relate to x and only depends on the true score y*(*t*). *Namely*,


(2) $$\begin{array}{l} \text{Pr}\left\{\tilde{y}\left(t\right)|y\left(t\right),x\left(t\right)\right\}=\text{Pr}\left\{\tilde{y}\left(t\right)|y\left(t\right)\right\},\;\forall t=1,...,T. \end{array}$$

Since the available data set $D_{T}$ has noisy score $\tilde{y}$(*t*), the estimated evaluator $\tilde{E}:X\to \mathbb{P}\left(Y\right)$ constructed by directly using $D_{T}$ will give the noisy probability distribution $\tilde{p}\left(\tilde{y}|x\right)$ instead of true probability distribution *p*(*y*|*x*), where the noisy probability distribution $\tilde{p}\left(\tilde{y}|x\right)$ is defined by


(3) $$\begin{array}{l} \tilde{p}\left(\tilde{y}|x\right):=\text{Pr}\left\{\tilde{y}|x\right\}. \end{array}$$

It is necessary to improve the robustness of the estimated evaluator and guide the evaluator to give the probability distribution near the true probability distribution even it is trained by using noisy data set $D_{T}$.

Then, the problem we address in this article is summarized as follows.

**Problem 1**. *Suppose that data set* $D_{T}$ *has been obtained from the market survey and Assumptions 1 and 2 hold. To find a robust evaluator* $E_{\text{r}}$ *by solving the following optimization problem*.


(4) $$\begin{array}{l} \underset{E}{\text{min}}\;𝔻\left(E,E_{\text{true}}\right). \end{array}$$

The challenging issue of solving Problem 1 is that the true probability distribution is unknown, which makes it difficult to find the direction of modifying the data set $D_{T}$ for training.

## 3. Robust robot image classification

### 3.1. Noisy transition matrix

With Assumption 2, it is able to establish the relationship between noisy label posterior $\tilde{p}\left(\tilde{y}|x\right)$ and the true label posterior *p*(*y*|*x*) as


(5) $$\begin{array}{l} \tilde{p}\left(\tilde{y}|x\right)=\overset{C}{\underset{y=1}{\sum }}p_{y}\left(\tilde{y}|y\right)p\left(y|x\right), \end{array}$$

where *p*_*y*_($\tilde{y}$|*y*): = Pr{$\tilde{y}$|*y*}. The true label posterior *p*(*y*|*x*) is essentially a vector-value function from X to [0, 1]^*C*^, which is written as


(6) $$\begin{array}{l} p\left(y|x\right)={\left[\text{Pr}\left\{y=1|x\right\},...,\text{Pr}\left\{y=C|x\right\}\right]}^{⊺}. \end{array}$$

The noisy label posterior $\tilde{p}\left(\tilde{y}|x\right)$ is also a vector-valued function written as


(7) $$\begin{array}{l} \tilde{p}\left(\tilde{y}|x\right)={\left[\text{Pr}\left\{\tilde{y}=1|x\right\},...,\text{Pr}\left\{\tilde{y}=C|x\right\}\right]}^{⊺}. \end{array}$$

On the other hand, *p*_*y*_($\tilde{y}$|*y*) is essentially a noisy transition matrix written as


(8) $$\begin{array}{l} p_{y}\left(\tilde{y}|y\right)=\left[\begin{array}{ccc} \text{Pr}\left\{\tilde{y}=1|y=1\right\} & ... & \text{Pr}\left\{\tilde{y}=C|y=1\right\}\\ ... & ... & ...\\ \text{Pr}\left\{\tilde{y}=1|y=C\right\} & ... & \text{Pr}\left\{\tilde{y}=C|y=C\right\} \end{array}\right]. \end{array}$$

Let ***T***_n_ be the notation of the noisy transition matrix instead of *p*_*y*_($\tilde{y}$|*y*) and $T_{\text{n}}\subseteq {\left[0,1\right]}^{C\times C}$ be the set of all possible ***T***_n_. Then, we can rewrite Equation (5) by


(9) $$\begin{array}{l} \tilde{p}\left(\tilde{y}|x\right)=T_{\text{n}}^{⊺}p\left(y|x\right). \end{array}$$

If the noisy transition matrix ***T***_n_ is available and *p*(*y*|*x*) is identifiable with $\tilde{p}\left(\tilde{y}|x\right)$ (every *p*(*y*|*x*) generate distinct $\tilde{p}\left(\tilde{y}|x\right)$), we can recover *p*(*y*|*x*) from $\tilde{p}\left(\tilde{y}|x\right)$ by using ***T***_n_ according to Equation (9). It is reasonable to assume that the true label posterior *p*(*y*|*x*) can be approximated by a parameterized model $\hat{p}\left(y|x,\theta \right)$ characterized by θ ∈ Θ. Namely, there exists one θ^*^ ∈ Θ such that $\hat{p}\left(y|x,\theta^{*}\right)=p\left(y|x\right)$ for every y∈Y and x∈X. Suppose that $\hat{p}\left(y|x,\theta \right)$ is differentiable with θ. Note that it is possible to represent $\hat{p}\left(y|x,\theta \right)$ by an expressive deep neural network characterized by θ.

For the learning objective, we adopt the expected Kullback-Leibler (KL) divergence, which is a standard objective. The expected KL divergence concerned here is constructed as follows:


(10) $$\begin{array}{l} L_{0,\text{true}}\left(\theta \right):={𝔼}_{x\sim p_{x}\left(x\right)}\left\{D_{\text{KL}}\left(\tilde{p}\left(\tilde{y}|x\right),T_{\text{n}}^{⊺}\hat{p}\left(y|x,\theta \right)\right)\right\}, \end{array}$$

where *p*_*x*_(*x*) is the probability density function defined on X. Note that *L*_0,true_(θ) has connections to the cross-entropy loss which is defined as


(11) $$\begin{array}{l} L_{\text{ce},\text{true}}\left(\theta \right):={𝔼}_{\left(x,\tilde{y}\right)\sim p\left(x,\tilde{y}\right)}\left\{-\log\left(T_{\text{n}}^{⊺}\hat{p}\left(y|x,\theta \right)\right)\right\}\\ \;=L_{0,\text{true}}\left(\theta \right)+H\left(\tilde{y}|x\right), \end{array}$$

where *H*($\tilde{y}$|*x*) is the conditional entropy, namely, the entropy of $\tilde{y}$ under *x*. Note that *H*($\tilde{y}$|*x*) is a constant with respect to θ and *L*_ce,true_(θ) is minimized if and only if *L*_0,true_(θ). If *L*_ce,true_(θ) is optimized, we can say that $T^{⊺}\hat{p}\left(y|x\right)=T^{⊺}p\left(y|x\right)$ and $\hat{p}\left(y|x\right)=p\left(y|x\right)$. For practice, although it is only possible to empirically estimate *L*_ce,true_(θ), we can still prove the asymptomatic convergence. Namely, as the number of samples increases, with probability 1, we have $T^{⊺}\hat{p}\left(y|x\right)\to T^{⊺}p\left(y|x\right)$ and $\hat{p}\left(y|x\right)\to p\left(y|x\right)$.

### 3.2. Classification without noisy transition matrix

In our case, both ***T***_n_ and *p*(*y*|*x*) are not available. Therefore, ***T***_n_ and *p*(*y*|*x*) are partially identifiable which means that there might exist multiple pairs of ***T***_n_ and *p*(*y*|*x*) with the same $\tilde{p}\left(\tilde{y}|x\right)$. Then, it is impossible to identify the true ***T***_n_ and *p*(*y*|*x*) without any further assumptions.

Let ${\hat{T}}_{\text{n}}\in T_{\text{n}}$ be an estimated noisy transition matrix. Then, the KL divergence is written as


(12) $$\begin{array}{l} L_{0}\left(\theta ,{\hat{T}}_{\text{n}}\right):={𝔼}_{x\sim p_{x}\left(x\right)}\left\{D_{\text{KL}}\left(\tilde{p}\left(\tilde{y}|x\right),{\hat{T}}_{\text{n}}^{⊺}\hat{p}\left(y|x,\theta \right)\right)\right\}. \end{array}$$

The cross-entropy loss is then revised as


(13) $$\begin{array}{l} L_{\text{ce}}\left(\theta ,{\hat{T}}_{\text{n}}\right):={𝔼}_{\left(x,\tilde{y}\right)\sim p\left(x,\tilde{y}\right)}\left\{-\log\left({\hat{T}}_{\text{n}}^{⊺}\hat{p}\left(y|x,\theta \right)\right)\right\}\\ \;=L_{0}\left(\theta ,{\hat{T}}_{\text{n}}\right)+H\left(\tilde{y}|x\right), \end{array}$$

In the case without noisy transition matrix, we should find θ and ${\hat{T}}_{\text{n}}$ to minimize $L_{\text{ce}}\left(\theta ,{\hat{T}}_{\text{n}}\right)$ or equivalently make $L_{0}\left(\theta ,{\hat{T}}_{\text{n}}\right)=0$. As the same with the case with noisy transition matrix, we can empirically obtain the estimation of $L_{\text{ce}}\left(\theta ,{\hat{T}}_{\text{n}}\right)$ based on samples of *x*, $\tilde{y}$-pairs and also optimize it by adjusting θ and ${\hat{T}}_{\text{n}}$. Although it is possible to ensure the convergence of ${\hat{T}}_{\text{n}}\hat{p}\left(y|x\right)$ to ***T***_n_*p*(*y*|*x*) with infinite sample size, the convergence of $\hat{p}\left(y|x\right)$ to *p*(*y*|*x*) may not be guaranteed if *p*(*y*|*x*) is not identifiable with $\tilde{p}\left(\tilde{y}|x\right)$ (Patrini et al., 2017).

Most of the existing methods adopt a two-step method. In the first step, the noisy transition matrix is estimated. Then, the estimated noisy transition matrix is used for neural network training. The estimation of the noisy transition matrix is based on the anchor points (Yu et al., 2018; Xia et al., 2022), which are defined as follows.

**Definition 1**. *An point x is called an anchor point for class i* = 1, ..., *C if* Pr{*y* = *i*|*x*} = 1.

For an anchor point *x* for class *i* = 1, ..., *C*, Equation (9) can be transformed to


(14) $$\begin{array}{l} \tilde{p}\left(\tilde{y}|x\right)=T_{\text{n}}p\left(y|x\right)=T_{\text{n},i}. \end{array}$$

Then, it is possible to estimate ***T***_n_ based on the estimation of $\tilde{p}\left(\tilde{y}|x\right)$ from the data with noisy labels. Note that the two-step method cannot be applied if anchor points can not be obtained from data clearly. However, estimating the noisy label posteriors has a dramatically worse overfitting issue than estimating the true label posteriors. Therefore, the estimation of the transition matrix suffers from the estimated noisy label distributions, which are not accurate and the performance deteriorates sharply.

Transition matrix estimation also suffers from poorly estimated noisy label posteriors and the performance deteriorates sharply.

### 3.3. Construction of equivalence class and partial order

It is necessary to deeply investigate the generating process of the class-conditional label noise and establish equivalence class and partial order for noisy transition matrix ***T***_n_. According to Zhang et al. (2021), partial order of the transition matrices led by the contraction property of the stochastic is shown to be able to find the true class posterior. Here, we summarize the idea of constructing equivalence class and partial order for noisy transition matrix, which is the theoretical basis of our proposed robust robot image classification.

The definition of transition matrix equivalence is as follows.

**Definition 2**. *Transition matrix equivalence is essentially an equivalence relation of a pair of matrices with an ordered product. We say* (***U***, ***V***) ~ (***U***′, ***V***′) *if **UV*** = ***U***′***V***′. *Besides, for a given matrix **W***, *the equivalence class associated with **W** is defined by*


(15) $$\begin{array}{l} \left[W\right]:=\left\{\left(U,V\right):UV=W\right\}. \end{array}$$

For noisy transition matrix ***T***_n_, there is also an equivalence class [*T*]. For a pair (***U***, ***V***) ∈ [***T***], we can form an optimal solution that minimizes (Equations 12, 13) by setting


(16) $$\begin{array}{l} {\hat{T}}_{\text{n}}=V, \end{array}$$

and


(17) $$\begin{array}{l} \hat{p}\left(y|x,\theta \right)=U^{⊺}p\left(y|x\right). \end{array}$$

Note that the potential optimal solutions are infinite and only (***I***, ***T***) is the true pair for our interest. Thus, it is important to investigate other conditions to direct us to the pair (***I***, ***T***) among infinite optimal solutions.

For any given optimal solution ${\hat{T}}_{\text{n}}$, $\hat{p}\left(y|x,\theta \right)$ of Equations (12) and (13), there exists a matrix ***U*** that satisfies $\hat{p}\left(y|x\right)=U^{⊺}p\left(y|x\right)$ if anchor points exists in data set $D_{T}$ for each class *i* (Zhang et al., 2021). Thus, we have the following assumption throughout the paper.

**Assumption 3**. *The obtained data set* $D_{T}$ *has at least one anchor point for each class i* = 1, ..., *C*.

With the absolute existence of anchor points as stated in Assumption 3, it is able to find a condition to break the transition matrix equivalence and obtain the desired pair (***I***, ***T***).

Let ***v*** and ***w*** be two categorical probabilities. For any ***v*** and ***w***, the total variation distance is defined by

**Definition 3**. *The total variation distance between two categorical probabilities is*


(18) $$\begin{array}{l} {𝔻}_{\text{TV}}\left(v,w\right):=\frac{||v-w|{|}_{1}}{2}, \end{array}$$

where ||·||_1_ is the ℓ_1_ norm.

Based on the theory of Markov chains (Moral et al., 2003), we have that ***v*** ↦ ***U***^⊺^***v*** is a contraction mapping with the total variation distance, namely,


(19) $$\begin{array}{l} {𝔻}_{\text{TV}}\left(U^{⊺}v,U^{⊺}w\right)\leq {𝔻}_{\text{TV}}\left(v,w\right),\forall v,w,\forall U\in T_{\text{n}}. \end{array}$$

With the above discussions, we can define partial order in the equivalence class [***T***_n_] as follows.

**Definition 4**. *The transition matrix partial order by the total variation distance is expressed as*


(20) $$\begin{array}{l} \left(U,V\right)≼\left(U^{\prime },v^{\prime }\right)\Leftrightarrow {𝔻}_{TV}\left(U^{⊺}v,U^{⊺}w\right)\leq {𝔻}_{TV}\left({U\prime }^{⊺}v,{U\prime }^{⊺}w\right),\forall v,w. \end{array}$$

Note that (***I***, ***T***) is the unique element for the greatest total variation (Zhang et al., 2021). Therefore, it is able to find (***I***, ***T***) by gradually increasing the total variation.

### 3.4. Total variation regularization

First, we define the expected total variation distance by


(21) $$\begin{array}{l} R\left(\theta \right):={𝔼}_{x_{1}\sim p\left(x\right)}{𝔼}_{x_{2}\sim p\left(x\right)}\left\{{𝔻}_{\text{TV}}\left({\hat{p}}_{1},{\hat{p}}_{2}\right)\right\}, \end{array}$$

where ${\hat{p}}_{i}:=\hat{p}\left(y|x=x_{i},\theta \right),i=1,2$. We summarize Theorem 2 in Zhang et al. (2021) here.

**Theorem 1**. *Suppose Assumption 3 holds. Let* ${\tilde{L}}_{0}\left(\theta ,{\hat{T}}_{\text{n}}\right)$ *and* $\tilde{R}\left(\theta \right)$ *be the empirical estimates of* $L_{0}\left(\theta ,{\hat{T}}_{\text{n}}\right)$ *and* *R*(θ) *by using data set* $D_{T}$, *respectively. Suppose* Θ *is compact. Let* $\tilde{\theta },{\tilde{T}}_{\text{n}}$ *be an optimal solution of the following optimization problem:*


(22) $$\begin{array}{l} \underset{\theta \in \Theta ,\;{\hat{T}}_{\text{n}}\in T_{\text{n}}}{\max}\;\tilde{R}\left(\theta \right)\;\text{s.t.}\;{\tilde{L}}_{0}\left(\theta ,{\hat{T}}_{\text{n}}\right)=0. \end{array}$$

*Then*, ${\tilde{T}}_{\text{n}}$ *is a consistent estimator of **T***_n_, *and* $\hat{p}\left(y|x,\tilde{\theta }\right)\to p\left(y|x\right)$ *with probability 1 as T* → ∞.

Theorem 1 claims that we can obtain a consistent estimate of noisy transition matrix by solving (Equation 22). The true probability distribution can also be obtained. Note that the constrained problem (Equation 22) can be solved by introducing Lagrangian


(23) $$\begin{array}{l} L\left(\theta ,{\hat{T}}_{\text{n}}\right):={\tilde{L}}_{0}\left(\theta ,{\hat{T}}_{\text{n}}\right)-\lambda \tilde{R}\left(\theta \right), \end{array}$$

where λ ∈ ℝ^+^ is a positive number that controls the importance of the regularization term. Therefore, the unconstrained problem (Equation 23) is called the optimization problem with total variation regularization.

### 3.5. Proposed algorithm

**Figure 2:**
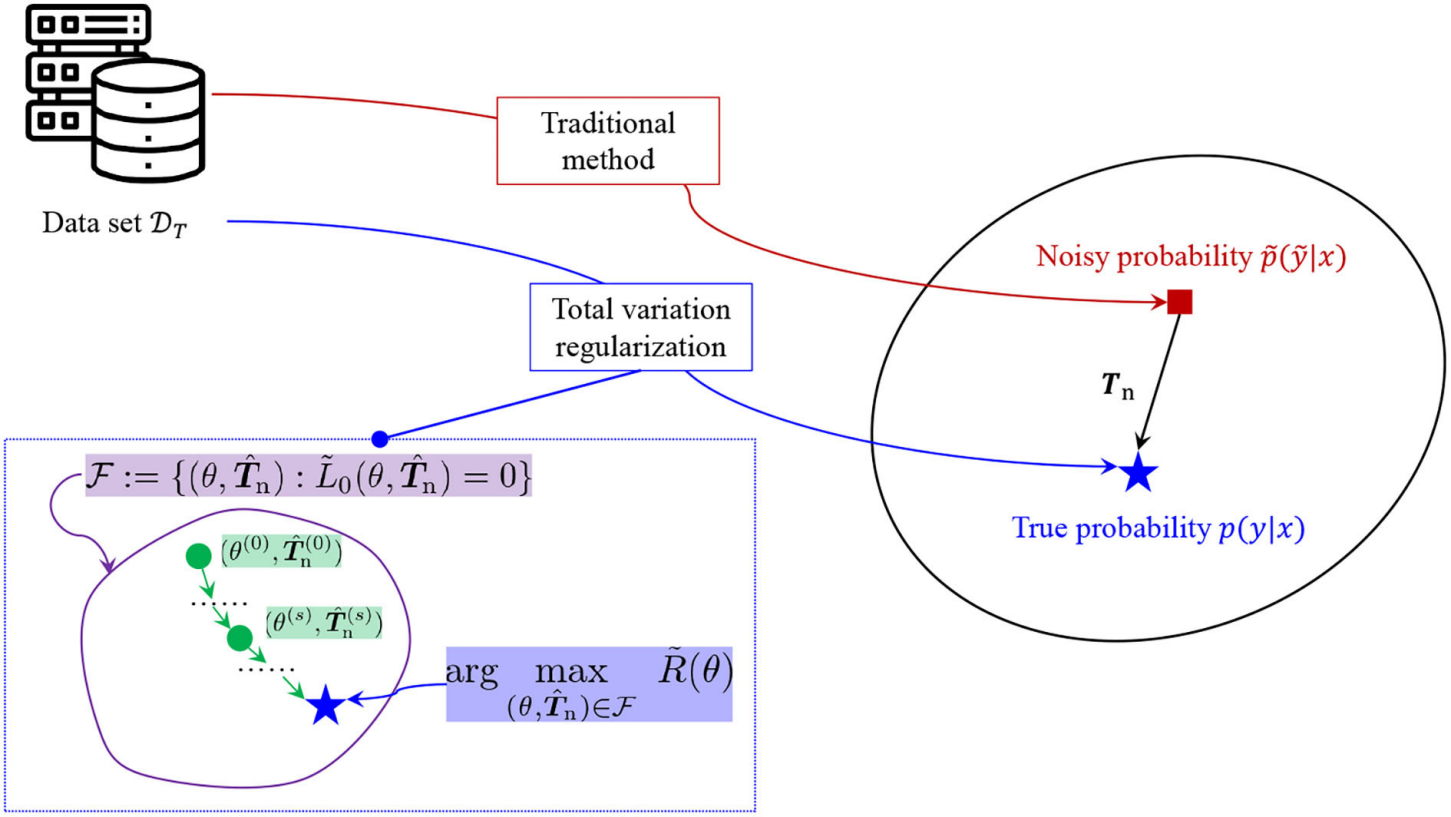
Concept of estimating the transition matrix and the probability distribution.

Then, we present the algorithms for estimating the transition matrix ***T***_n_ and also the probability distribution simultaneously. The concept of the simultaneous estimation is illustrated in Figure 2.

Note that $L\left(\theta ,{\hat{T}}_{\text{n}}\right)$ is differentiable with respect to ${\hat{T}}_{\text{n}}$. Therefore, it is possible to use gradient-based optimization to find a local minimum for ${\hat{T}}_{\text{n}}$ and $\tilde{p}\left(y|x,\theta \right)$. To make sure that ${\hat{T}}_{\text{n}}\in T_{\text{n}}$, we can use softmax to an unconstrained matrix and optimize $L\left(\theta ,{\hat{T}}_{\text{n}}\right)$ by stochastic gradient descent. The proposed algorithm is adapted from Adam algorithm (Kingma and Ba, 2015) and is summarized as follows.

**Remark 1**. Algorithm 1 solves Problem 1 under Assumptions 1, 2, and 3 by making $𝔻\left(E,E_{\text{true}}\right)\to 0$ with probability 1 if *S, T* → ∞.

**Algorithm 1:**
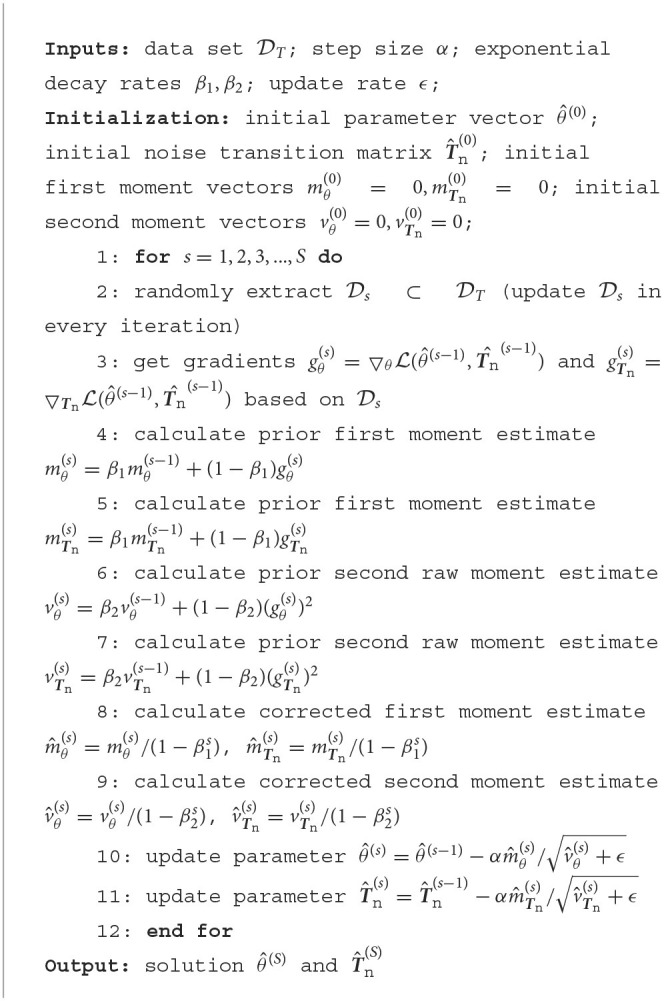
One-step algorithm for estimating ${\hat{T}}_{\text{n}}$ and $\tilde{p}\left(y|x,\theta \right)$ based on stochastic gradient descent.

The proposed method can be regarded as a perfect version of the clustering-based method. In the clustering algorithm-based method, some of the data with wrong labels are forbidden. However, there is no existing clustering algorithm-based method that can make sure that all the data with wrong labels are forbidden and all the data with correct labels remain, which can be ensured by the proposed method if Assumptions 1, 2, and 3 hold.

### 3.6. Implementation of the proposed robust robot classification

**Figure 3:**
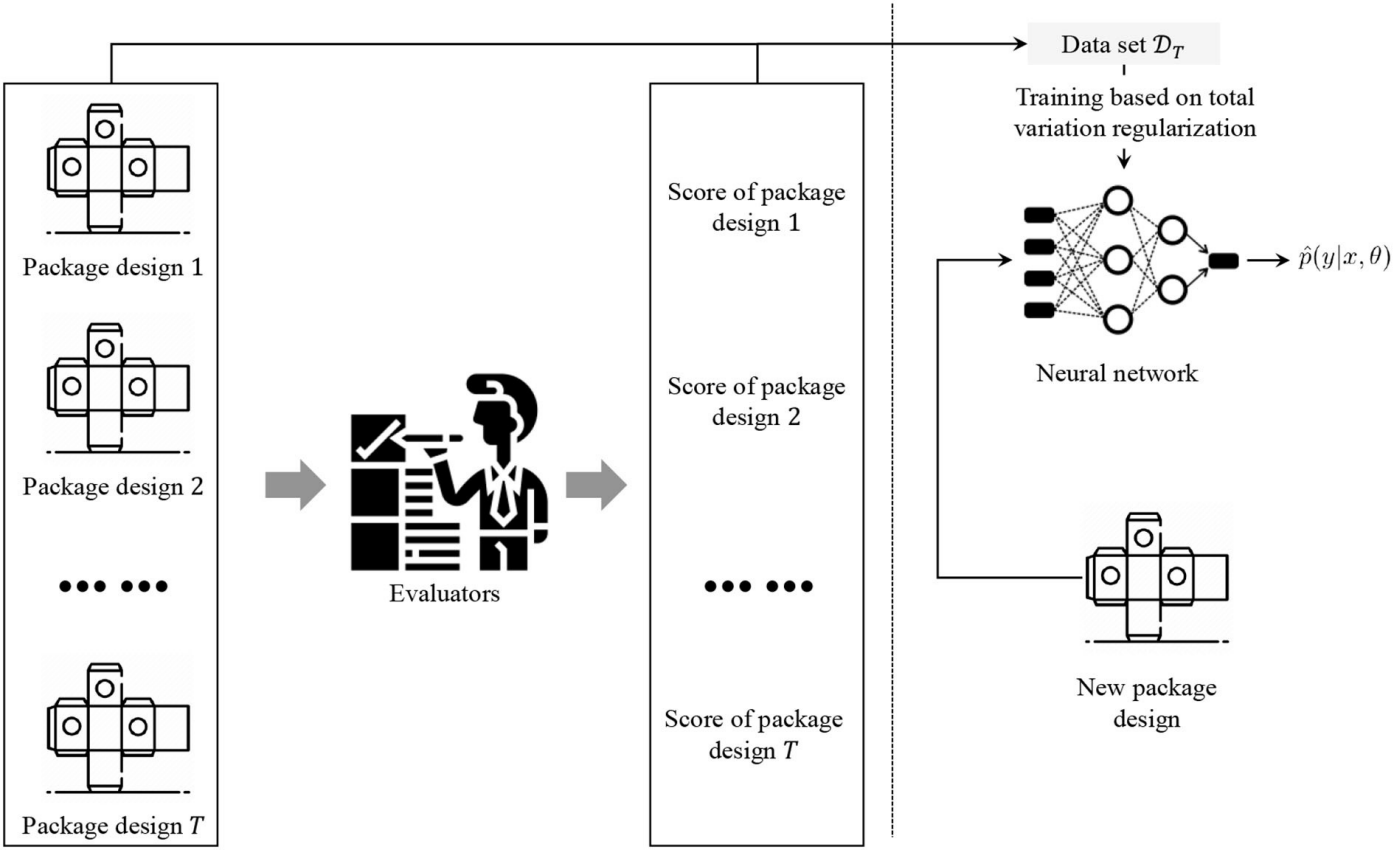
Process of implementation of the proposed robust robot classification for evaluating package design.

The process of implementing the proposed robust robot classification for evaluating package design is illustrated in Figure 3. First, we construct the data set $D_{T}$ to train the neural networks. A total of *T* package designs are collected with different levels. Then, several evaluators are asked to give labels to each package design. The evaluators make the labels from the viewpoint of a customer. Then, total variation regularization is applied to data set $D_{T}$ to train neural networks that output the estimated probability distribution. For any given package design, with the outputted probability distribution by the trained neural networks, the score or the classification will be determined by


(24) $$\begin{array}{l} i\left(x\right)=\arg\underset{i}{\max}\hat{p}\left(y=i|x,\theta \right). \end{array}$$

## 4. Validations

In this section, the results of experimental data are presented to validate the effectiveness of the total variation regularization-based robust robot classification method. The results show that the proposed method can improve the accuracy of the classification even using noisy labels compared to the traditional method.

### 4.1. Experimental data set and methods

A total of 5,000 different package designs have been collected. All package designs are categorized into six classes, namely, *C* = 5. In every class, there are 1,000 samples. The categorization has been implemented by some experienced evaluators. In this experiment, we regard the label given by experienced evaluators as real ones and the noisy labels are generated by using the following kinds of label noises:

- (Pair.) defines the pair flipping noise for labeling, which is introduced by Han et al. (2018);
- (Symm.) represents the symmetric noise for labeling, which is introduced by Patrini et al. (2017);
- (Rand.) is random noise generated by Dirichlet distribution mixing with the identity matrix.

**Figure 4:**
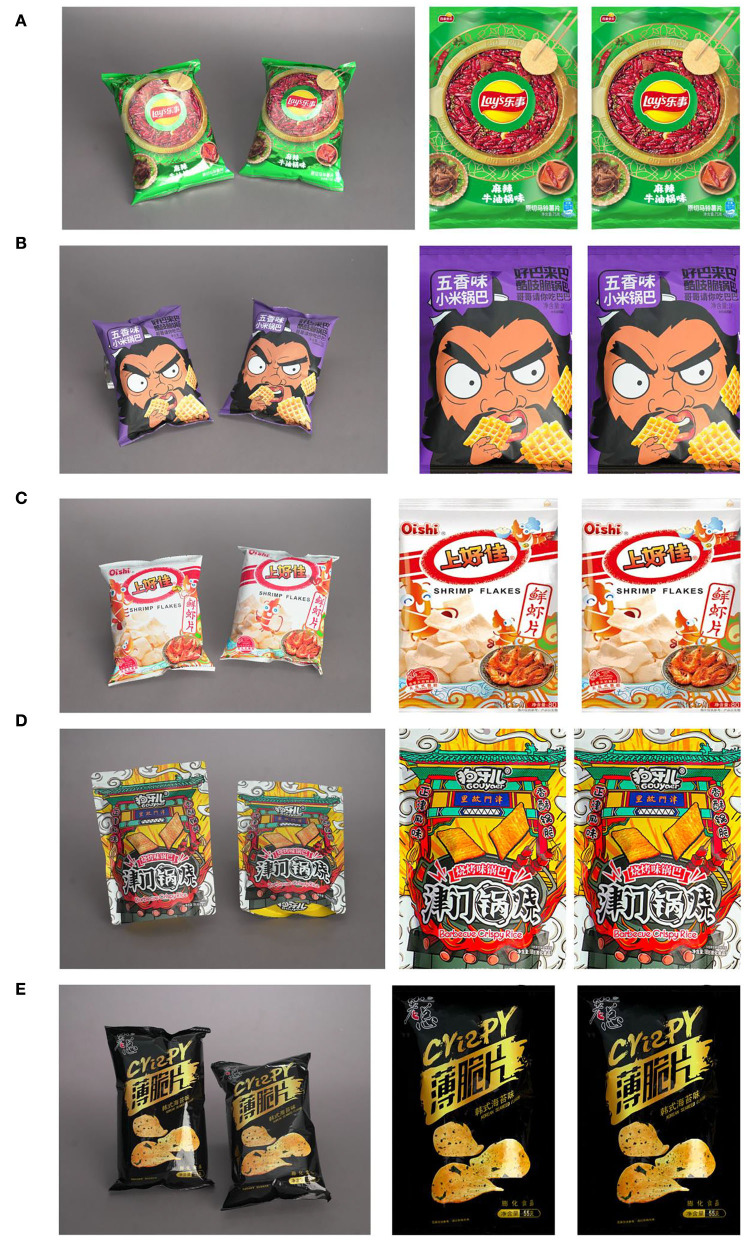
Examples of experimental data set: **(A)** one example in class 1 (with score 1); **(B)** one example in class 2 (with score 2); **(C)** one example in class 3 (with score 3); **(D)** one example in class 4 (with score 4); **(E)** one example in class 5 (with score 5).

Figure 4 shows five examples of experimental data sets from five different classes. It is reasonable to use the labels given by experienced evaluators since experienced evaluators can give relatively precise labels according to their rich experience in the market and package design. In addition, 70% of the data set has been used for training and the rest is for testing. The data for training have noisy labels and the data for testing are with true labels.

In this validation, sequential convolutional neural network (CNN) has been used for classifier models.

### 4.2. Results and discussions

**Figure 5:**
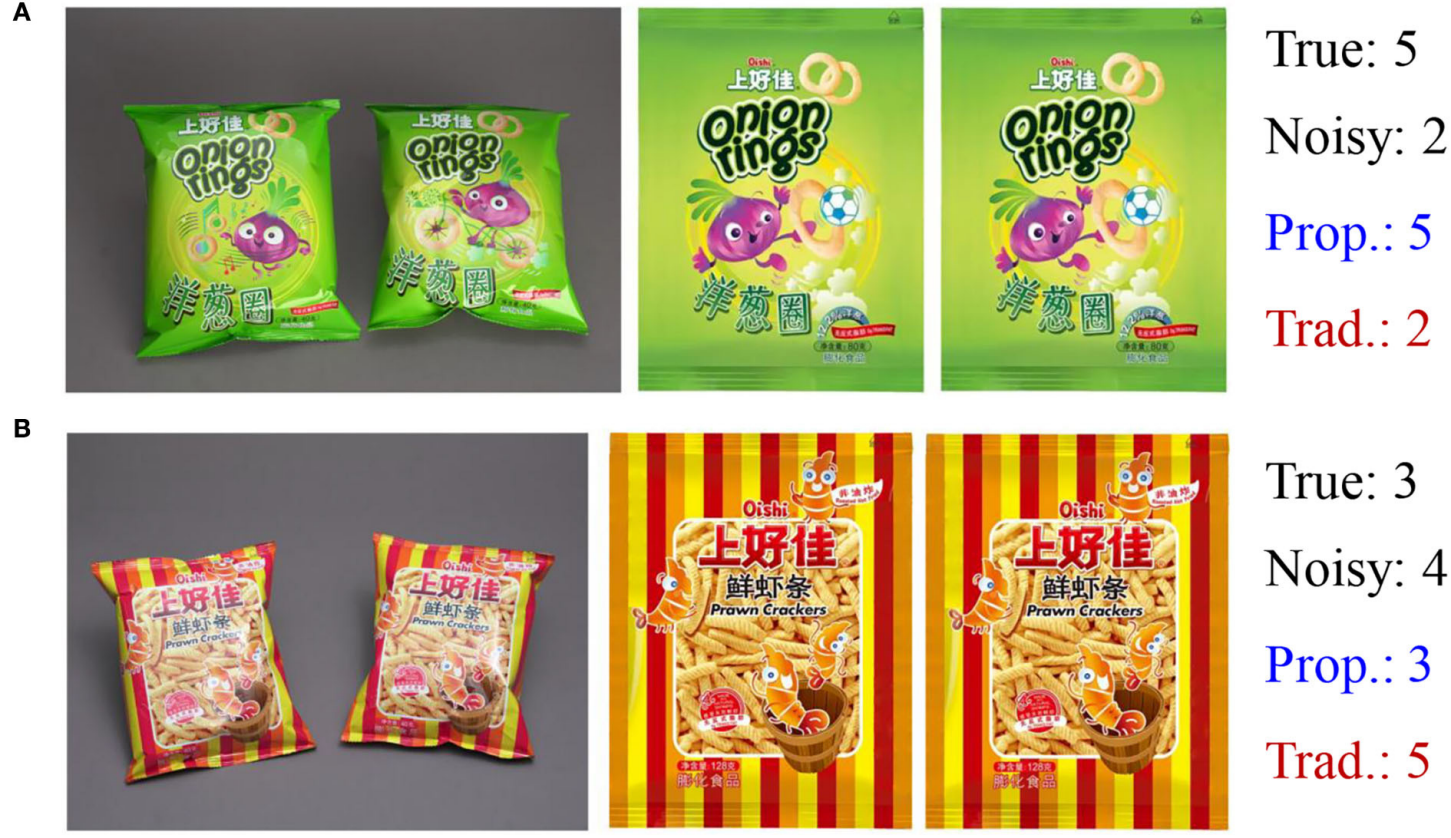
Illustrative examples of the results: **(A)** one design with a score of 5 in the test set; **(B)** one design with a score of 3 in the test set.

Figure 5 gives two illustrative examples of the validation results with random noise whose noise rate is 35%. (Prop.) is short for the proposed method. (Trad.) is short for the traditional method in which noisy labels are used for training as true labels. The proposed method gives results that are consistent with the true labels despite using noisy labels. On the other hand, the traditional method gives results different from the true labels. Since true labels and noisy labels both exist, the traditional method gets confused and sometimes gives results that are not consistent with both.

In Table 1, the test accuracy is reported. The traditional method uses noisy labels for training. The baseline is obtained by using the true labels for training. It is obvious that the proposed method outperforms the traditional method in terms of accuracy and shows very close accuracy with the baseline. As the percentage of noise rate increases, the accuracy of the traditional method decreases dramatically while the proposed method only has a very slight deterioration in accuracy. For the types of noise, even with random noise, the proposed method still gives very accurate results. The above results show the effectiveness of the proposed method.

**Table 1:** Accuracy (%) on test set.

**Noise rate**	**Methods**	**Symm**.	**Pair**	**Rand**
15%	Traditional	94.11	87.24	43.71
	Proposed	99.42	99.37	98.02
	Baseline	99.67	99.62	99.77
25%	Traditional	91.98	83.57	36.22
	Proposed	99.38	99.27	97.66
	Baseline	99.67	99.62	99.77
35%	Traditional	90.65	81.73	32.98
	Proposed	99.27	99.15	95.32
	Baseline	99.67	99.62	99.77
45%	Traditional	88.91	78.31	29.047
	Proposed	99.26	99.12	94.29
	Baseline	99.67	99.62	99.77
55%	Traditional	87.29	77.12	26.97
	Proposed	99.21	99.05	93.91
	Baseline	99.67	99.62	99.77

## 5. Conclusion

In this paper, a novel robust robot image classification method for package design evaluation has been introduced. The proposed method can give high accuracy in classification even with noisy labels in the training process. In the proposed method, the loss function for training is total variation regularization whose optimal solution is consistent with the true probability distribution of the labels. The proposed method has been validated by experimental data and it exhibits outperformed accuracy compared to the traditional method. With the proposed robot image classification, it is possible to establish a close-loop package design process, in which the designers can use the robot to help them improve their design.

## Data availability statement

The original contributions presented in the study are included in the article/supplementary material, further inquiries can be directed to the corresponding author/s.

## Author contributions

The author confirms being the sole contributor of this work and has approved it for publication.
